# Evaluation of ecological consequences on the global distribution of *Staphylococcus aureus* Rosenbach 1884 due to climate change, using Maxent modeling

**DOI:** 10.1038/s41598-025-87534-2

**Published:** 2025-04-03

**Authors:** Monerah S. M. Alqahtani, Gasser Shahin, Ibrahim T. I. Abdelalim, Sameh M. H. Khalaf

**Affiliations:** 1 Biology Department, Faculty of Science, King Khalid University, 61413 Abha, Saudi Arabia; 2 Faculty of Biotechnology, October University for Modern Sciences & Arts (MSA University), 6th October City, 12566 Egypt

**Keywords:** Climate change, Opportunistic pathogen, Bioclimatic variables, Habitat suitability, Maxent modeling, Biogeography, Climate-change ecology, Ecological modelling, Microbial ecology, Microbiology

## Abstract

*Staphylococcus aureus* is a primary cause of many infections in humans, and its rising prevalence and drug resistance are serious public health concerns. While there is evidence that climate change can influence the distribution and abundance of microbial species, the precise effects on *S. aureus* are not well characterized. The purpose of this study is to predict the potential influence of climate change on the global distribution of *Staphylococcus aureus* in 2050 and 2070 using GIS and Maxent modeling. *S. aureus* occurrence data was acquired from global databases and coupled with bioclimatic variables to simulate current and future habitat suitability under several climate change scenarios (RCP 2.6 and 8.5). The Maxent modeling approach was used to forecast geographical patterns of *S. aureus* distribution, providing insights into locations that may see increased prevalence of this essential species as a result of climate change. The study’s findings can be used to inform public health measures and focused surveillance activities aimed at reducing the burden of *Staphylococcus aureus* infection.

## Introduction

Many human infections, such as those affecting the skin and soft tissues, pneumonia, bacteremia, and endocarditis, are caused by the Gram-positive, coagulase-positive bacterium *Staphylococcus aureus*^1,2^. Up to 30% of the population usually has this commensal bacterium on their skin and in their nasal passages^3^. A variety of illnesses, especially in those with weakened immune systems, can be caused by *S. aureus* when it acts as an opportunistic pathogen^4^. *Staphylococcus aureus*’s alarmingly high disease burden and prevalence rates warrant serious attention. There is substantial evidence that its antimicrobial resistance is on the rise, and experts project that it will be responsible for 10 million fatalities by the year 2050. It may be challenging to eradicate *Staphylococcus aureus* in clinical settings, and it is a leading cause of infections of the skin and soft tissues, surgical wounds, and bloodstreams^2^.

Due to their inability to be effectively treated by currently available antibiotics, antibiotic-resistant *Staphylococcus aureus* strains like methicillin-resistant *Staphylococcus aureus* (MRSA) have emerged as a major public health concern^5,6^. When it comes to designing methods to lessen the impact of *S. aureus* on human health, knowing what factors affect its distribution and prevalence is essential. Instances of community-acquired *Staphylococcus aureus* infection are known to spike during periods of high rainfall in East Malaysia, according to certain empirical research^7^. Despite strong empirical evidence linking climate change to shifts in the prevalence of *Staphylococcus aureus* infections, the exact mechanisms by which these shifts take place are still largely unknown. The prevalence of *Staphylococcus aureus* exhibits significant variability among diverse demographics and geographical areas. About 30% of the human population is asymptomatically colonized by S. *aureus*, mostly in the nasal cavity, presenting a risk for subsequent infections, particularly in susceptible populations such as surgery patients and individuals with weakened immune systems^2^. The incidence of MRSA in community settings is increasing, with some studies suggesting that in certain regions, up to 50% of *S. aureus* isolates may be MRSA^5^. The incidence of *Staphylococcus aureus* bacteremia varies from 10 to 30 instances per 100,000 person-years in industrialized regions, with notable discrepancies observed in nonindustrialized areas^1^. The rising incidence of antibiotic-resistant strains and the related healthcare difficulties highlight the necessity for improved surveillance, efficient infection control strategies, and responsible antibiotic stewardship to alleviate the effects of *Staphylococcus aureus* infections.So, we’re going to make the case that, in a climate-changed world, we need to know how this phenomenon could affect the spread of particular diseases if we want to see long-term improvements in health care.

Many microbial species, including infectious diseases, may see shifts in distribution and frequency as a result of climate change^7,8^. Various microorganisms may see changes to their distribution and abundance as a result of changes in their optimal habitats brought about by changes in temperature, precipitation patterns, and other environmental variables^9,10^. The dynamics of microbial communities can be further impacted by human behavior changes brought about by climate change, such as population shifts and changes in agricultural methods^11,12^.

*Staphylococcus aureus* infections are influenced by various environmental and climatic conditions that affect their epidemiology, distribution, and abundance. Warmer temperatures can enhance the growth and survival of *Staphylococcus aureus*, particularly in urban environments where heat islands are prevalent, leading to increased transmission rates in densely populated areas^1^. Elevated humidity levels contribute to the persistence of the bacteria in the environment, facilitating their survival on surfaces and in the air, which increases the likelihood of infection transmission^7^. Additionally, the prevalence of *Staphylococcus aureus* infections often varies with the seasons; certain strains may become more common during warmer months when outdoor activities increase the risk of skin injuries, serving as entry points for the bacteria^3^. Socio-economic factors also play a significant role, as environmental conditions interact with issues like overcrowding and access to healthcare. Areas with limited healthcare resources may experience higher rates of infections, particularly in populations living in close quarters where transmission is more likely. Furthermore, climate change and extreme weather events, such as floods and heat waves, can disrupt sanitation and healthcare infrastructure, creating conditions favorable for bacterial proliferation. Understanding these multifaceted interactions is crucial for developing effective public health strategies to mitigate the impact of *Staphylococcus aureus* infections^10^.

Some of the most useful tools for assessing how climate change can affect the distribution of different species, including microbes, are Geographical Information Systems (GIS) and species distribution modeling approaches like MaxEnt. Several previous studies were using GIS to evaluate the status of pathogenic and nonpathogenic microorganisms. The effectiveness of this method is the clear prediction of how these species distribution will be in the near and far future^13,14^. These methods can be used to find places where the *S. aureus* infection is likely to be more common, which in turn can guide public health initiatives and specific monitoring programs.

This study sets out to do just that by examining how several climate change scenarios could affect the distribution and prevalence of *Staphylococcus aureus* in the years 2050 and 2070. This effort will utilize GIS and MaxEnt modeling to shed light on the temporal and spatial patterns of *S. aureus* spread. This knowledge can help in devising ways to reduce the public health hazards linked to this significant pathogen.

## Results

### Model assessment and contribution of environmental variables

**Fig. 1:**
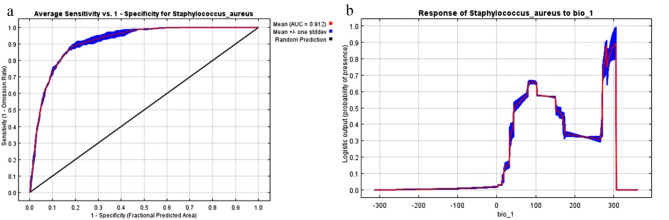
(**a**) the Area under the Curve graph showing the value of 0.91 which indicates the accuracy of the model; (**b**) the response curve of Annual mean temperature bio_1.

The MaxEnt model employed to forecast the appropriateness of habitats for *Staphylococcus aureus* exhibited exceptional performance, as seen by the elevated Area Under the Curve (AUC) value of 0.91 (Fig. 1a) and the Total Seasonal Score (TSS) of 0.71. These measures validate the model’s exceptional accuracy in capturing the environmental interactions of the species.

The Jackknife test was performed to evaluate the impact of the five most relevant bioclimatic factors. The findings indicated that bio1, representing the Annual Mean Temperature, had the highest level of significance, accounting for 58.6% of the contribution. This was followed by bio17, representing the Precipitation of the Driest Quarter with 26, then bio11, representing the Mean Temperature of the Warmest Quarter, contributed 6.5%. bio 6, which represents the Minimum Temperature of the Coldest Month and bio14, representing the Precipitation of the Driest Month, contributed 4.8%. and 4.1% respectively, which made a smaller but still noteworthy contribution.

The response curves (Fig. 1b) also indicated that the optimal temperature range for *Staphylococcus aureus* is between two different ranges around 10 °C and around 30 °C which may represent situation in winter and summer. This corresponds with the discovery that the Annual Mean Temperature (bio1) is the most significant factor in determining the suitability of the pathogen’s habitat. The knowledge gained from analyzing the main factors influencing the environment and the specific environmental conditions preferred by *Staphylococcus aureus* is crucial for comprehending the possible spread and occurrence of this bacterium under various climate change scenarios.

### Current situation of ***Staphylococcus aureus*** distribution based on climatological Maxent modeling

**Fig. 2:**
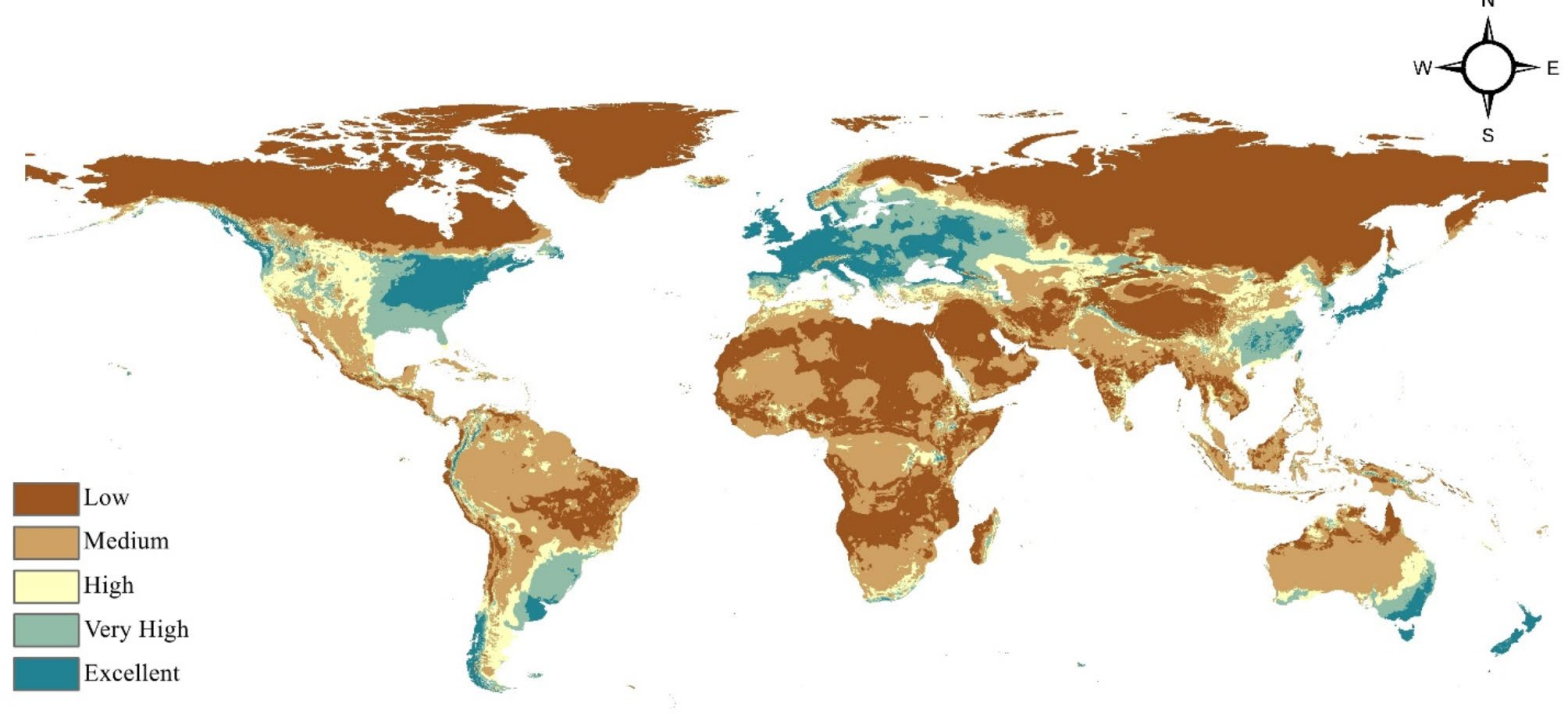
Current distribution model of *Staphylococcus aureus*.

The model developed illustrates the present worldwide distribution and the suitability of habitats for *Staphylococcus aureus* (Fig. 2), a prevalent and consequential bacterial disease. Extensive areas worldwide, especially in the middle Amazon basin, central Africa, and parts of middle Asia, are classified as having limited potential for the spread of this bacteria. On the other hand, a considerable part of the globe, which includes a large portion of North America, Europe, and East Asia, is categorized as having moderate suitability. In these regions, the environmental conditions and resources are likely suitable for the survival and growth of the subject, but they may not be the most ideal areas for its widespread distribution.

Regions exhibiting significant appropriateness for *Staphylococcus aureus* are rather restricted, encompassing portions of the eastern United States, western Europe, the Middle East, and certain areas of Southeast Asia. These regions are most likely to provide the optimal conditions for the bacterium to flourish. A limited number of locations, mainly in the southern United States, the United Kingdom, and parts of the Middle East, are classified as having exceptional suitability, offering the ideal combination of factors required for the bacterium to achieve its utmost capacity for growth and dispersion. The comprehensive categorizations depicted in the map indicate that *Staphylococcus aureus* is widely distributed worldwide, with notable variations in its adaptability across different places. This knowledge can contribute to public health initiatives, focused surveillance, and proactive steps to mitigate the potential hazards and consequences of this significant bacterial pathogen.

### Future situation of *Staphylococcus aureus* distribution based on climatological change scenario

The provided models present a thorough depiction of the anticipated alterations in the worldwide habitat suitability for *Staphylococcus aureus* under several climate change scenarios, as simulated by the MRI-CGCM3 General Circulation Model.

**Fig. 3:**
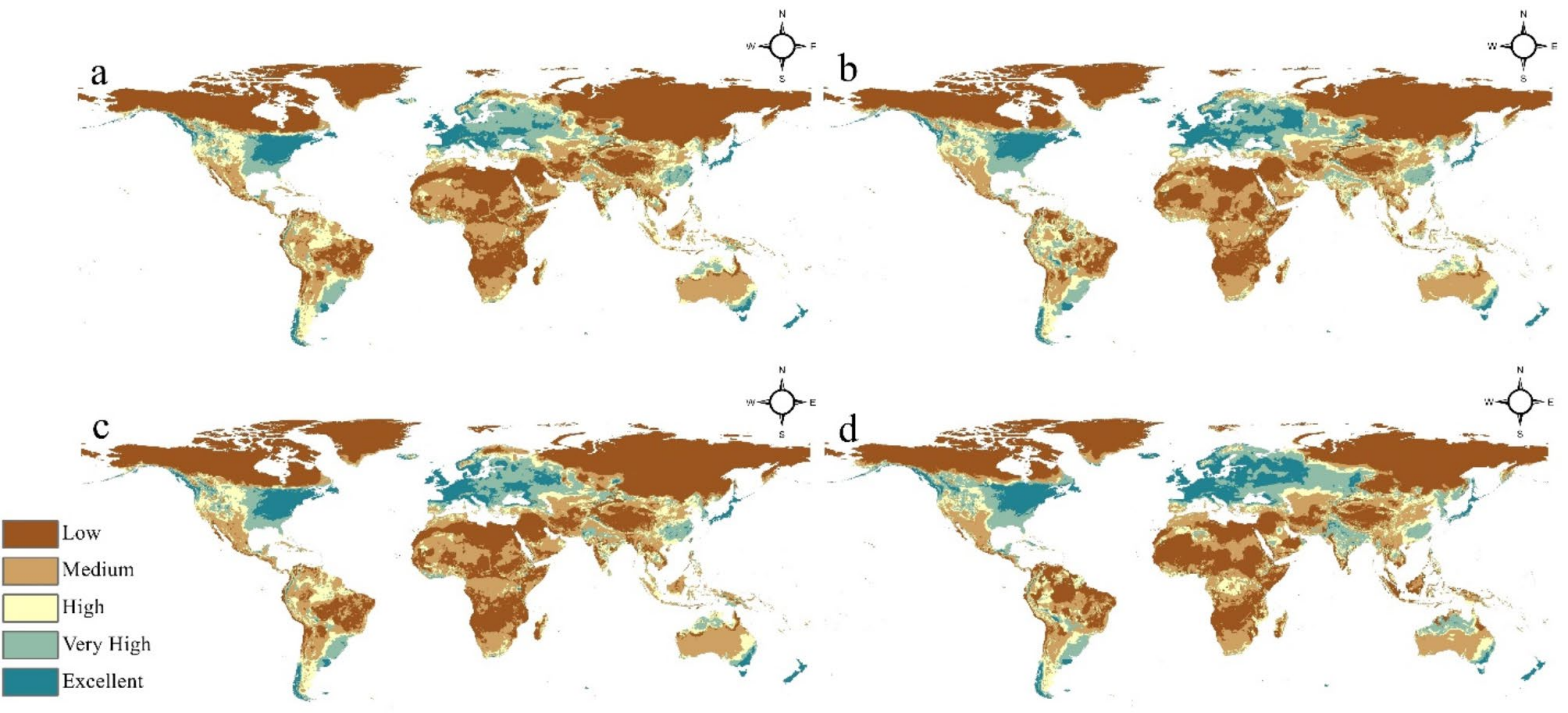
Future distribution model of *Staphylococcus aureus*: (**a**) 2050 at RCP 2.6; (**b**) 2050 at RCP 8.5; (**c**) 2070 at RCP 2.6 and (**d**) 2070 at RCP 8.5.

The maps labeled Fig. 3a,b depict the anticipated suitable conditions for the year 2050, based on the RCP 2.6 and RCP 8.5 scenarios, respectively. According to the RCP 2.6 scenario (a), there is an increase in the appropriateness of certain locations, mainly in North America, Europe, and Asia, with a higher likelihood of being suitable or extremely suitable. Nevertheless, the general worldwide distribution pattern remains rather consistent with the present circumstances, albeit with little increases in appropriateness observed in different locations.

On the other hand, the RCP 8.5 scenario (b), which depicts a more severe trajectory of greenhouse gas emissions, demonstrates a more noticeable change in the worldwide suitable landscape by 2050. Extensive regions in North America, Europe, and Asia are currently categorized as having a high or very high appropriateness for *Staphylococcus aureus*. Furthermore, there has been an observable increase in the regions that exhibit exceptional adaptability, particularly in the southern United States and certain areas in the Middle East.

Looking ahead to the forecasts for 2070, the lower maps, Fig. 3c,d, show the possible suitable conditions based on the RCP 2.6 and RCP 8.5 scenarios, respectively. According to the RCP 2.6 scenario (c), there is a continued increase in the number of sites globally that are highly suitable or very highly suitable for certain purposes. In particular, regions like North America and Europe are seeing outstanding levels of appropriateness. Nevertheless, the RCP 8.5 scenario (d) for 2070 depicts a substantially modified landscape, characterized by a more extensive and noticeable rise in adaptability worldwide. Extensive areas in North America, Europe, Asia, and certain parts of Africa are now classified as having high, very high, or exceptional appropriateness for the growth and spread of *Staphylococcus aureus* due to significant changes in habitat range and favorable environmental circumstances.

These projections emphasize the need to take into account the possible effects of climate change on the spread and occurrence of this important bacterial pathogen. The global patterns of suitability are expected to change significantly in the next few decades, especially under the more extreme emissions scenario.

**Fig. 4:**
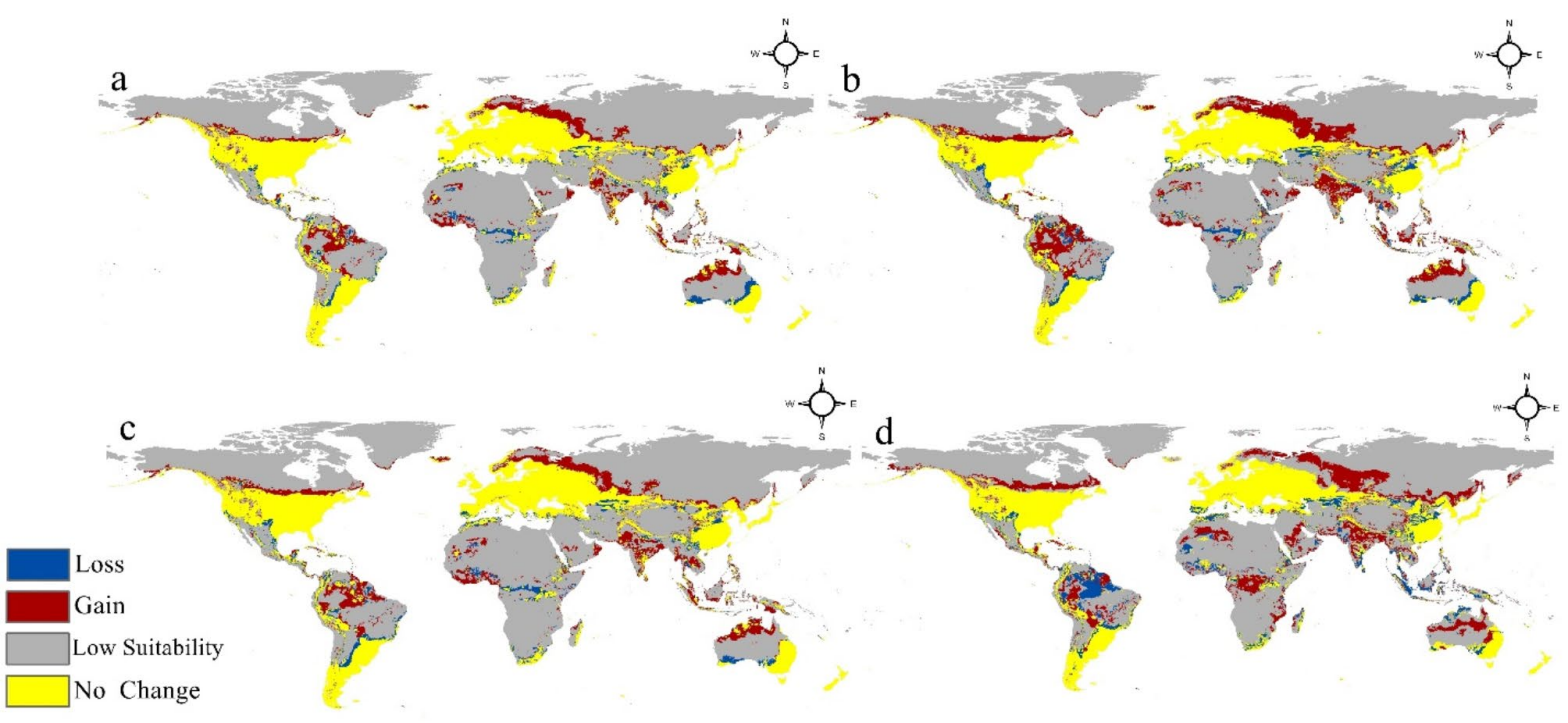
Calibration maps for the impact of climate change on *Staphylococcus aureus* distribution: (**a**) 2050 at RCP 2.6; (**b**) 2050 at RCP 8.5; (**c**) 2070 at RCP 2.6 and (**d**) 2070 at RCP 8.5.

The calibration maps illustrate regions of both “Gain” and “Loss” in the appropriateness for the bacterium (Fig. 4). The red shaded zones represent places where the appropriateness for *Staphylococcus aureus* is projected to rise, potentially resulting in an extension of its habitat and dispersion. In contrast, the blue-colored patches indicate locations where the appropriateness is projected to decline, leading to a reduction in suitable habitat for the pathogen. These subsequent forecasts emphasize additional changes in the worldwide distribution, with more prominent locations experiencing both increased and decreased suitability, as well as regions where suitability is anticipated to remain largely stable, as denoted by the yellow coloring.

The calibration maps serve as a great tool for comprehending the possible effects of climate change on the occurrence and spread of *Staphylococcus aureus*, a major public health issue. The precise spatial data can provide valuable insights for focused surveillance, evaluation of potential risks, and the formulation of measures to counteract the projected variations in the bacterium’s habitat appropriateness across various global locations.

### Two dimensional niche analysis

**Fig. 5:**
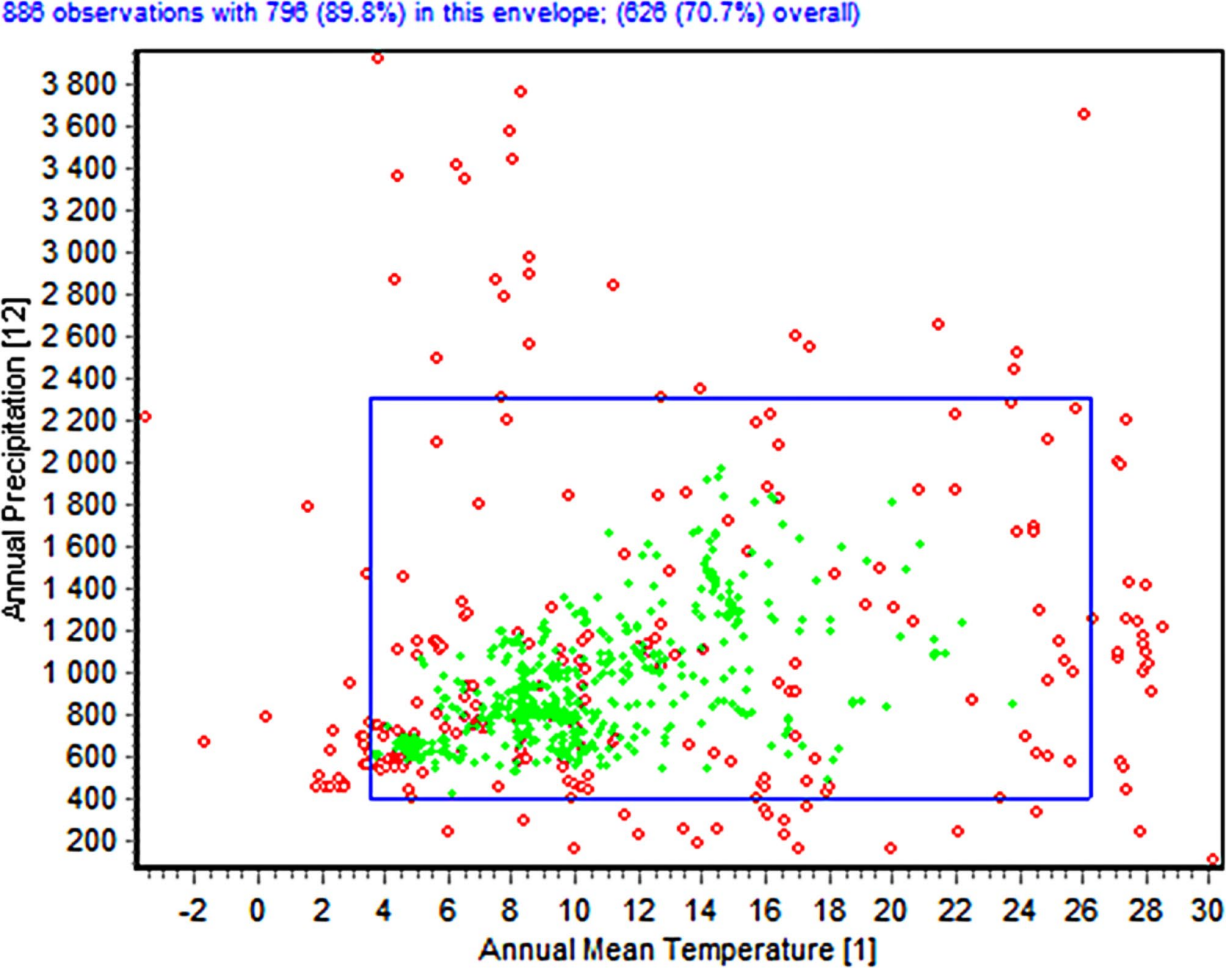
The two-dimensional niche between Annual Temperature (bio 1) and Annual Precipitation (bio 12) for *Staphylococcus aureus*.

The scatter plot of two-dimensional niche analysis (Fig. 5) provides vital insights regarding the pathogen’s habitat range and environmental preferences. The data points demonstrate that the pathogen has a remarkably wide range of tolerance for yearly mean temperatures, ranging from approximately − 2 °C to over 30 °C. This indicates that the pathogen possesses a high degree of adaptability and is capable of flourishing in a wide range of thermal conditions. Moreover, the data points are distributed across a broad spectrum of precipitation levels, suggesting that this bacterium is capable of thriving in both humid and arid conditions. This is consistent with the assertion that the disease has a “cosmopolitan distribution” and is capable of thriving in both arid, hot regions and damp, chilly conditions. The data points are distributed in a scattered manner, encompassing a wide range of temperature and precipitation levels. This demonstrates that the pathogen is capable of occupying many ecosystems. The pathogen’s ecological flexibility is a significant factor in its global dispersion and capacity to adapt to different environmental settings.

The large range of ecological niches that the pathogen can exploit, as found in this investigation, indicates its great environmental tolerance. This adaptability may contribute to its effective establishment and proliferation in various places worldwide. Overall, the dimensional niche analysis emphasizes the impressive ability and wide range of habitats that this disease can adapt to, indicating its capacity to flourish in different climate conditions and geographic areas. This is presumably the reason for its reported widespread dispersion.

## Discussion

This study’s findings offer valuable insights into how climate change could potentially affect the worldwide distribution and frequency of *Staphylococcus aureus*. The MaxEnt models used in this analysis indicate that the appropriate habitat for any biological entity is expected to increase or decrease in the future under both moderate (RCP 2.6) and high (RCP 8.5) emission scenarios^15^.

The present distribution pattern suggests that *S. aureus* is extensively spread around the world, with significant prevalence in densely populated areas, such as some regions in North America, Europe, and Asia. This is consistent with the established ecological role of *S. aureus* as a prevalent bacteria that lives harmoniously on the skin and mucous membranes of a substantial number of people^16^.

According to the future estimates based on the RCP 2.6 and RCP 8.5 scenarios, the habitat that is favorable for *S. aureus* is expected to grow by 2050 and continue expanding by 2070. The locations seeing the most significant growth in habitat appropriateness are situated in higher latitudes, specifically in northern North America, northern Europe, and northern Asia. Climate change is expected to cause higher temperatures and increased precipitation in these locations, perhaps leading to more favorable conditions for the spread of *S. aureus*.

The proliferation of favorable habitat for *S. aureus* is worrisome, since it may result in heightened disease prevalence and burden in these areas. *Staphylococcus aureus* is a prominent culprit behind a range of infections, such as skin and soft tissue infections, pneumonia, bacteremia, and endocarditis. The expansion of favorable habitat can promote the dissemination of both drug-sensitive and drug-resistant strains of *S. aureus*, including methicillin-resistant *S. aureus* (MRSA), which presents substantial public health concerns^17^.

The projected rise in *S. aureus* habitat suitability seems to be primarily influenced by the anticipated alterations in temperature and precipitation patterns. The MaxEnt models indicated that bio_1, bio_6, bio_11, bio_14 and bio_17 affect the distribution of *S. aureus*. These variables are anticipated to see substantial modifications in the climate change scenarios, perhaps leading to more favorable conditions for the spread of this bacterium.This is combatable with several previous habitat suitability publications which deal with future prediction^18–20^.

Other factors, such as alterations in human behavior and activities, can also impact the expansion of suitable habitat for *S. aureus*^21^. Instances such as population migrations, urbanization, and changes in farming methods can all impact the dynamics of microbial communities, including the spread and occurrence of *S. aureus*^22^.

The results of this research are consistent with the effects that climate change has been shown to have on the distribution and abundance of other microbial species, including the opportunistic fungal disease *Aspergillus fumigatus*, commonly referred to as black mold. Like *S. aureus*, research indicates that *A. fumigatus*’s suitable habitat is expected to grow as a result of climate change, especially in areas with higher temperatures and more precipitation^23^. This implies that changes in the climate could affect the distribution and quantity of different microbes, both harmful and non-pathogenic.

As a limitation of the current study, future studies should take into account the socioeconomic and behavioral characteristics to gain a more thorough knowledge of the intricate interplay between climate change, environmental factors, and the epidemiology of *S. aureus*. The thing that can not done on a global scale with absence of future data for populations, epidemiology and socioeconomic impact. The findings of this study can be used to establish targeted strategies to reduce the possible public health problems connected with the spread of *Staphylococcus aureus*. These tactics could include: Improving monitoring and early warning systems. The identification of areas at higher risk of rising *S. aureus* prevalence can help guide the adoption of improved surveillance and monitoring measures. This can help to spot outbreaks earlier and conduct interventions more quickly^24^; Strengthening antimicrobial stewardship programs: Given the growing problem of antibiotic resistance, it is critical to strengthen antimicrobial stewardship programs in healthcare and the community. This can help to maintain the efficacy of current antimicrobial therapies and slow the spread of drug-resistant *S. aureus* strains^25^.

Finally, the findings of this study underscore the need for continued research on the complex interactions between climate change, microbial ecology, and public health. Exploring the impacts of climate change on a wider range of microorganisms, as well as the potential synergistic effects, can inform more comprehensive strategies to address the challenges posed by infectious diseases in a changing climate.

## Conclusion

*Staphylococcus aureus* is a leading cause of various infections in humans, and the increasing prevalence and antibiotic resistance of this bacterium is a major public health concern. While there is evidence that climate change can influence the distribution and prevalence of microbial species, the specific impacts on *S. aureus* remain poorly understood. This study aims to analyze the potential impact of climate change on the global distribution of *Staphylococcus aureus* in 2050 and 2070 using GIS and MaxEnt modeling. Occurrence data for *S. aureus* was obtained from global databases and combined with bioclimatic variables to model the current and future habitat suitability under different climate change scenarios (RCP 2.6 and RCP 8.5). The MaxEnt modeling approach was used to predict the spatial patterns of *S. aureus* distribution, providing insights into areas potentially at risk of increased prevalence of this important pathogen as a result of climate change.

The findings of this study demonstrate that climate change is likely to significantly impact the global distribution of *Staphylococcus aureus*, with potential increases in habitat suitability in certain regions by 2050 and 2070. These projections can inform public health interventions and targeted surveillance efforts to mitigate the impact of *S. aureus* infections. As climate change continues to shape the environmental conditions worldwide, understanding the ecological factors driving the distribution of this opportunistic pathogen is crucial for developing effective strategies to combat its spread and the associated public health risks.

## Material and methods

### Occurrence data

**Fig. 6:**
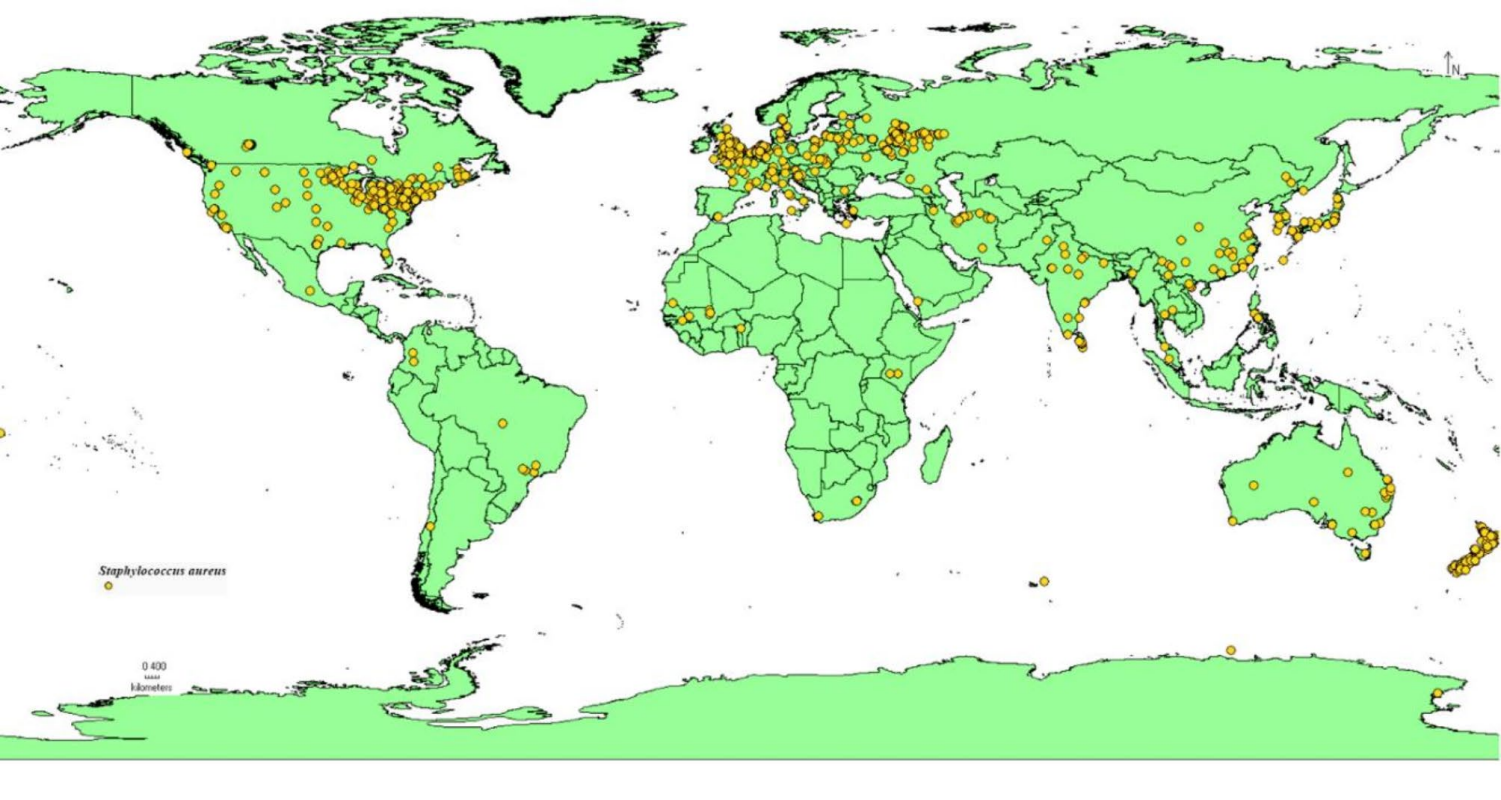
The occurrence records derived from the final dataset used for modeling the habitat suitability of *Staphylococcus aureus*.

The Global Biodiversity Information Facility database (www.gbif.org) provided the records for *Staphylococcus aureus*^26^. 11,186 occurrence points were first collected in order to model this bacterium distribution. Three primary procedures were used to filter the data. First, remove points lacking latitudes and longitudes; next, remove duplicate records; and finally, use ArcGIS v. 10.3 (SDM toolbox: SDM tools; Universal tools—Spatially rarefy occurrence data) to perform spatial rarefaction based on a 50 km distance^27^. To simulate the possible distribution of *S. aureus* in the future, 1,053 unique sites were converted to CSV format (Fig. 6). Around the world, the bacterium is mostly found in areas with dense populations of people^28^.

### Environmental data

A total of nineteen bioclimatic variables, which include temperature and precipitation data, were acquired from the WorldClim database (www.worldclim.org). The variables possess a spatial resolution of 2.5 arc-minutes (equivalent to 5 square kilometres) and encompass monthly climatic data from the years 1950 to 2000, which were gathered by forecast stations.

In the current prediction model, bioclimatic variables 8–9 and 18–19 were not included since their resolution was affected by spatial inconsistencies^29,30^. The remaining fifteen variables were transformed into ASCII format using ArcGIS v. 10.3. In order to exclude any association between variables, Pearson’s correlation coefficient was employed, with a threshold of r2 ≥ |0.8|. In addition, the SDM toolbox feature in ArcGIS v. 10.3 was utilized to decrease multicollinearity. This universal tool allowed for the exploration of climate data and the removal of highly correlated variables^31^. Five bioclimatic factors of utmost importance were chosen for further analysis.

The future prediction model obtained a specific set of variables from the WorldClim database for the time periods 2050 and 2070, based on representative concentration pathways (RCPs) 2.6 and 8.5. RCPs are estimates of radiative forcing components that are used as input for climate models. RCP 2.6 represents the scenario with the lowest emissions, while RCP 8.5 represents the scenario with the highest emissions^32^. The layers were utilised in the subsequent model after being converted to ASCII format using ArcGIS v. 10.3.

### Modeling approach

The current and future distribution of *Staphylococcus aureus* was simulated using the MaxEnt software package v. 3.4.1^33^. MaxEnt predicts the presence of a species by merging occurrence records with background data from environmental variables in the study area^34,35^. The places are ranked based on their similarity to present conditions, ranging from 0 (unsuitable) to 1 (suitable)^34^. The MaxEnt algorithm has shown effective in accurately predicting the potential geographic range of many bacterial species that are of significance in the fields of medicine and economics, both at local and global scales^36^.

The occurrence records were partitioned into two sets, with 75% of the data allocated for training the MaxEnt models and the remaining 25% for testing. This division enabled us to analyze the model’s performance on separate data and assess its predictive capability. A frequently used method in ecological modelling research is to adopt a split of 75% for one option and 25% for another^33^. The iteration count was set to 500, which corresponds to the number of times the MaxEnt method modifies model parameters in order to maximize the chance of occurrence data, given the environmental factors. Choosing the right number of iterations is essential for ensuring that the model reaches convergence and stability^33^. The background points were set to 10,000, representing the environmental conditions in the research region. Background points are randomly generated pseudo-absences that are utilized to establish the range of environmental circumstances that the species can inhabit^34^. To achieve a comprehensive coverage of the study area while still being computationally feasible, a total of 10,000 background points were selected^27^. In order to enhance the performance of the model, we employed a technique called 10-fold cross-validation^27^. This method involves dividing the data into various subsets, training the model on one subset, and evaluating its performance on the remaining data. Through iterative execution of this procedure, we derived an approximation of the model’s performance on various subsets of data^34^. Using ArcGIS version 10.3, regions were categorized into five groups according to their habitat suitability: inappropriate, low, medium, high, and extremely high^36,37^. This categorization enabled us to comprehend and convey the results of the model in a significant and readily comprehensible way.

### Model assessment

The models that were produced were assessed for performance using the True Skill Statistics (TSS) and the Area Under the Curve (AUC) of the Receiver Operating Characteristics (ROCs)^38,39^. To evaluate the overall model fit, the AUC values were employed. AUC values were classified as having a poor fit if they were less than 0.5 and as having a good fit if they were higher than 0.75^31^.

To offer a more thorough evaluation of model accuracy, the TSS was also computed. TSS values vary from − 1 to + 1, with values close to 0 indicating a poor relationship and values close to 1 indicating a strong link between the model and the distribution (Hosni et al. 2022). By accounting for both commission and omission mistakes, the TSS measure offers a more impartial assessment of the prediction performance of the model. We were able to get a strong assessment of the model’s capacity to precisely forecast the present and prospective future distribution of Staphylococcus aureus by utilizing both the AUC and TSS measures.

### Two-dimensional niche

We used Diva-GIS software to examine the two-dimensional niche of *Staphylococcus aureus*. Two important environmental variables—annual mean temperature (bio_1) and yearly precipitation (bio_12)—were the focus of the Envelope test^27^. We were able to evaluate the variables’ range and suitability for the species through this test^30^.

## Data Availability

All the data are included in the manuscript.

## References

[1] Lowy, F. D. Staphylococcus aureus infections. *N. Engl. J. Med.* **339**(8), 520–532. 10.1056/NEJM199808203390806 (1998). 9709046 10.1056/NEJM199808203390806

[2] Tong, S. Y., Davis, J. S., Eichenberger, E., Holland, T. L. & Fowler, V. G. Staphylococcus aureus infections: Epidemiology, pathophysiology, clinical manifestations, and management. *Clin. Microbiol. Rev.* **28**(3), 603–661. 10.1128/cmr.00134-14 (2015). 26016486 10.1128/CMR.00134-14 PMC4451395

[3] Kluytmans, J., van Belkum, A. & Verbrugh, H. Nasal carriage of Staphylococcus aureus: Epidemiology, underlying mechanisms, and associated risks. *Clin. Microbiol. Rev.* **10**(3), 505–520. 10.1128/cmr.00134-14 (2015). 10.1128/cmr.10.3.505 PMC172932 9227864

[4] Archer, G. L. Staphylococcus aureus: A well-armed pathogen. *Clin. Infect. Dis.* **26**(5), 1179–1181. 10.1086/520289 (1998). 9597249 10.1086/520289

[5] Chambers, H. F. The changing epidemiology of Staphylococcus aureus? *Emerg. Infect. Dis.* **7**(2), 178. 10.3201/eid0702.010204 (2001). 11294701 10.3201/eid0702.010204 PMC2631711

[6] David, M. Z. & Daum, R. S. Community-associated methicillin-resistant Staphylococcus aureus: Epidemiology and clinical consequences of an emerging epidemic. *Clin. Microbiol. Rev.* **23**(3), 616–687. 10.1128/cmr.00081-09 (2010). 20610826 10.1128/CMR.00081-09 PMC2901661

[7] Lim, K. T. Genetic and phenotypic characterisation of clinical methicillin-resistant staphylococcus aureus in a Malaysian hospital/Lim King Ting (Doctoral dissertation, University of Malaya). (2012).

[8] Lafferty, K. D. The ecology of climate change and infectious diseases. *Ecology* **90**(4), 888–900. 10.1890/08-0079.1 (2009). 19449681 10.1890/08-0079.1

[9] Burge, C. A. et al. Climate change influences on marine infectious diseases: Implications for management and society. *Annual Rev. Mar. Sci.* **6**, 249–277. 10.1146/annurev-marine-010213-135029 (2014). 10.1146/annurev-marine-010213-135029 23808894

[10] Semenza, J. C. & Menne, B. Climate change and infectious diseases in Europe. *Lancet. Infect. Dis*. **9**(6), 365–375. 10.1016/S1473-3099(09)70104-5 (2009). 19467476 10.1016/S1473-3099(09)70104-5

[11] Kilpatrick, A. M. & Randolph, S. E. Drivers, dynamics, and control of emerging vector-borne zoonotic diseases. *Lancet* **380**(9857), 1946–1955. 10.1016/S0140-6736(12)61151-9 (2012). 23200503 10.1016/S0140-6736(12)61151-9 PMC3739480

[12] Patz, J. A. et al. Unhealthy landscapes: Policy recommendations on land use change and infectious disease emergence. *Environ. Health Perspect.* **112**(10), 1092–1098. 10.1289/ehp.6877 (2004). 15238283 10.1289/ehp.6877 PMC1247383

[13] Elith, J. et al. Novel methods improve prediction of species’ distributions from occurrence data. *Ecography* **29**(2), 129–151. 10.1111/j.2006.0906-7590.04596.x (2006).

[14] Peterson, A. T. *Mapping Disease Transmission risk: Enriching Models Using Biogeography and Ecology* (Johns Hopkins University, 2014).

[15] Sharma, M. K., Ram, B. & Chawla, A. Ensemble modelling under multiple climate change scenarios predicts reduction in highly suitable range of habitats of Dactylorhiza Hatagirea (D. Don) Soo in Himachal Pradesh, western Himalaya. *South. Afr. J. Bot.* **154**, 203–218. 10.1016/j.sajb.2022.12.026 (2023).

[16] Obanda, B. A. Molecular epidemiology of staphylococcus aureus from hospital patients, HIV positive and negative abattoir workers and animals in Busia County, Kenya (Doctoral dissertation, University of Nairobi). (2021).

[17] Elmaidomy, A. H. et al. Antimicrobial potentials of natural products against multidrug resistance pathogens: A comprehensive review. *RSC Adv.* **12**(45), 29078–29102 (2022). 36320761 10.1039/d2ra04884a PMC9558262

[18] Alkhalifah, D. H. M., Damra, E., Khalaf, S. M. & Hozzein, W. N. Biogeography of black mold aspergillus Niger: global situation and future perspective under several climate change scenarios using MaxEnt modeling. *Diversity* **14**(10), 845. 10.3390/d14100845 (2022).

[19] Cohen, S. D. Estimating the climate niche of sclerotinia sclerotiorum using maximum entropy modeling. *J. Fungi*. **9**(9), 892. 10.3390/jof9090892 (2023). 10.3390/jof9090892 PMC10532795 37755000

[20] Alkhalifah, D. H. M., Damra, E., Melhem, M. B. & Hozzein, W. N. Fungus under a changing climate: Modeling the current and future global distribution of fusarium oxysporum using geographical information system data. *Microorganisms* **11** (2), 468. 10.3390/microorganisms11020468 (2023). 36838433 10.3390/microorganisms11020468 PMC9967672

[21] Park, S. & Ronholm, J. *Staphylococcus aureus* in agriculture: Lessons in evolution from a multispecies pathogen. *Clin. Microbiol. Rev.* **34**(2), 10–1128 (2021). 10.1128/CMR.00182-20 PMC7950364 33568553

[22] Steadmon, M. Drivers of Staphylococcus aureus Dynamics and Survival in Recreational Waters (Doctoral dissertation, University of Hawai’i at Manoa). (2024).

[23] Rocchi, S. et al. Impacts of climate change on the rise in Aspergillus diseases in Europe. *Mycopathologia* **183**(1), 191–204 (2018).

[24] Knox, J., Uhlemann, A. C. & Lowy, F. D. *Staphylococcus aureus* infections: Transmission within households and the community. *Trends Microbiol.* **23**(7), 437–444. 10.1016/j.tim.2015.03.007 (2015). 25864883 10.1016/j.tim.2015.03.007 PMC4490959

[25] Tenover, F. C. & Goering, R. V. Methicillin-resistant Staphylococcus aureus strain USA300: Origin and epidemiology. *J. Antimicrob. Chemother.* **64**(3), 441–446. 10.1093/jac/dkp241 (2009). 19608582 10.1093/jac/dkp241

[26] GBIF.org. GBIF Occurrence Download 22 July (2024). 10.15468/dl.gh6e7p

[27] Hosni, E. M. et al. Radwan. Modeling the Potential Global Distribution of Honeybee Pest, Galleria mellonella under changing Climate insects, **13**(5), 484. (2022). 10.3390/insects13050484 10.3390/insects13050484 PMC9143048 35621818

[28] Chen, C. J. & Huang, Y. C. New epidemiology of *Staphylococcus aureus* infection in Asia. *Clin. Microbiol. Infect.* **20**(7), 605–623 (2014). 24888414 10.1111/1469-0691.12705

[29] Escobar, L. E., Lira-Noriega, A., Medina-Vogel, G. & Peterson, A. T. Potential for spread of the white-nose fungus (pseudogymnoascus destructans) in the Americas: use of Maxent and NicheA to assure strict model transference. *Geospat. Health*. **9**(1), 221–229. 10.4081/gh.2014.19 (2014). 25545939 10.4081/gh.2014.19

[30] Hosni, E. M., Nasser, M. G., Al-Ashaal, S. A., Rady, M. H. & Kenawy, M. A. Modeling current and future global distribution of *Chrysomya bezziana* under changing climate. *Sci. Rep.* **10**(1), 4947. 10.1038/s41598-020-61962-8 (2020). 32188920 10.1038/s41598-020-61962-8 PMC7080715

[31] Nasser, M., El-Hawagry, M. & Okely, M. Environmental niche modeling for some species of the genus Anthrax Scopoli (Diptera: Bombyliidae) in Egypt, with special notes on St. Catherine protected area as a suitable habitat. *J. Insect Conserv.* **23**(5), 831–841. 10.1007/s10841-019-00174-6 (2019).

[32] Van Vuuren, D. P. et al. The representative concentration pathways: An overview. *Clim. Change*. **109**, 5–31 (2011).

[33] Phillips, S. J., Dudík, M. & Schapire, R. E. Maxent software for modeling species niches and distributions. (2023). https://biodiversityinformatics.amnh.org/open_source/maxent/

[34] Kumar, S., Neven, L. G., Zhu, H. & Zhang, R. Assessing the global risk of establishment of Cydia Pomonella (Lepidoptera: Tortricidae) using CLIMEX and MaxEnt niche models. *J. Econ. Entomol.* **108**(4), 1708–1719. 10.1093/jee/tov166 (2015). 26470312 10.1093/jee/tov166

[35] Zacarias, D. A. Global bioclimatic suitability for the fall armyworm, *Spodoptera frugiperda* (Lepidoptera: Noctuidae), and potential co-occurrence with major host crops under climate change scenarios. *Clim. Change*. **161**(4), 555–566 (2020).

[36] Hosni, E. M., Al-Khalaf, A. A., Nasser, M. G., ElShahed, S. M. & Alashaal, S. A. *Locusta migratoria* (L.)(Orthoptera) in a warming world: unravelling the ecological consequences of climate change using GIS. *Biodiversity Data Journal*, **12** (2024). 10.3897/BDJ.12.e115845 10.3897/BDJ.12.e115845 PMC10933582 38481856

[37] Wang, Z., Piche-Choquette, S., Lauzon, J., Ishak, S. & Kembel, S. W. Modeling plant-microbe interactions with species distribution models. 10.1101/2024.07.12.603317 (2024). 2024-07.40093181

[38] Al-Khalaf, A. A., Nasser, M. G. & Hosni, E. M. Global Potential Distribution of *Sarcophaga dux* and *Sarcophaga haemorrhoidalis* under Climate Change. Diversity, **15**(8), 903. (2023). 10.3390/d15080903

[39] Zhang, K., Yao, L., Meng, J. & Tao, J. Maxent modeling for predicting the potential geographical distribution of two peony species under climate change. *Sci. Total Environ.* **634**, 1326–1334. 10.1016/j.scitotenv.2018.04.112 (2018). 29710632 10.1016/j.scitotenv.2018.04.112

